# Eponyms in andrology

**DOI:** 10.1186/2051-4190-24-7

**Published:** 2014-04-15

**Authors:** Khalid Al Aboud, Daifullah Al Aboud, Sameer Munshi, Ali assiry Halawi

**Affiliations:** King Faisal Hospital, P.O Box 5440, Makah, 21955 Saudi Arabia; Taif University, Taif, Saudi Arabia

**Keywords:** Andrology, Eponym, Nomenclature, Male fertility, andrologie, éponyme, nomenclature, infécondité masculine

## Abstract

Andrology is the study of male reproductive health, its associated medicines, and biology, including functions and diseases that are specific to men, especially with regard to the reproductive organs. This concise report discusses the eponyms that are encountered in andrological literature.

## Résumé

Eponymes en Andrologie

L’andrologie est l’étude de la santé reproductive masculine, de ses médicaments et de sa biologie, ainsi que des fonctions et maladies qui sont spécifiques aux hommes, tout particulièrement celles qui concernent les organes reproducteurs. Ce rapport succinct discute les éponymes rencontrés dans la littérature andrologique.

Andrology is the scientific discipline that covers men’s health issues—essentially the male counterpart to the study of female reproductive health, or gynecology. However, unlike gynecology, andrology remains a less pervasive and extensively studied medical discipline. Increasing research and care in this specialty will ensure proper management of medical conditions that are related to men’s health.

As in other specialties, most of the nomenclature in male reproductive medicine and male sexuality are descriptive and are derived from Latin and Greek. The male genitalia were called “testes”, likely from the Latin word “testis,” which originally meant “witnesses”, because they provided evidence of virility [1], whereas sperm is derived from the Greek word (σπέρμα) sperma (meaning “seed”).

Prefixes from these ancient languages have been used to describe various conditions of semen and sperm—for example, oligospermia (few spermatozoa in semen) and globozoospermia for globe-headed spermatozoa.

Abbreviations are also widely used in andrological literature, such as in vitro fertilization/intracytoplasmic sperm injection (IVF/ICSI), intracytoplasmic morphologically selected sperm injection (IMSI), and testicular sperm extraction (TESE) [2].

Eponyms are frequently encountered in andrology, but many doctors have no information about the origin or history of these eponyms.

In this commentary, we present select eponyms in andrology [3–8] (Table 1).

**Table 1:** **Selected eponyms in Andrology literature**

Eponyms in Andrology	Remarks
Kartagener syndrome [3, 4]	This is an older term for primary ciliary dyskinesia (PCD). PCD is a rare disease, predominantly inherited as an autosomal recessive, with ciliary dysfunction leading to impaired mucociliary clearance and chronic airway infection. Situs inversus totalis occurs in ~50% of PCD patients. In male patients, sperm flagella might show impairments in or lack the ability to swing, which ultimately results in male infertility. Manes Kartagener (1897-1975) (Figure 1) was a Swiss internist.
Klinefelter's syndrome [5]	A chromosomal disorder in which there is at least one extra X chromosome to a standard human male, causing a total of 47 chromosomes instead of 46. Principal effects include hypogonadism and sterility. It is named for Harry Fitch Klinefelter, Jr (1912-1990) (Figure 2), an American physician.
Leydig cells [6]	Histologically, they are adjacent to the seminiferous tubules in the testicle. They produce testosterone. They are named for Franz Leydig (1821–1908) (Figure 3), a German anatomist. Leydig discovered them in 1850.
Peyronie’s disease [7]	Also known as chronic inflammation of the tunica albuginea (CITA), it is a connective tissue disorder involving the growth of fibrous plaques in the soft tissue of the penis, causing erectile dysfunction. It is named after François Gigot de La Peyronie (1678-1747) (Figure 4), the first surgeon to Louis XV.
Priapism [7]	In this condition, the erect penis or clitoris does not return to its flaccid state, despite the absence of physical and psychological stimulation, within 4 hours. The name comes from the Greek god Priapus, a fertility god often represented with a disproportionately large and permanent erection.
	The acronym ASPEN syndrome was proposed for the association of sickle cell disease, priapism, exchange transfusion, and neurological events.
Sertoli cells [8]	Histologically, they are part of a seminiferous tubule. Their main function is to nourish developing sperm cells. They are named for Enrico Sertoli (1842–1910) (Figure 5), an Italian physiologist who discovered them while studying medicine at the University of Pavia, Italy.

**Figure 1:**
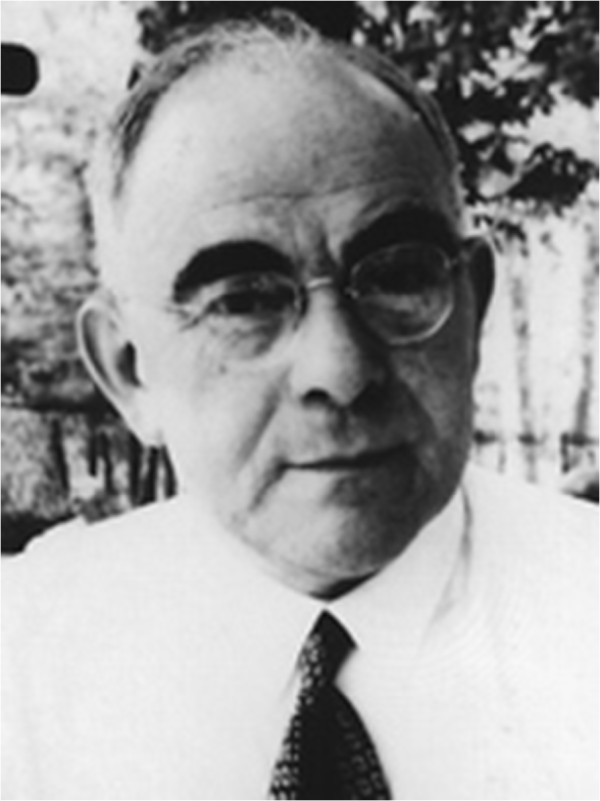
**Manes Kartagener (1897–1975).**

**Figure 2:**
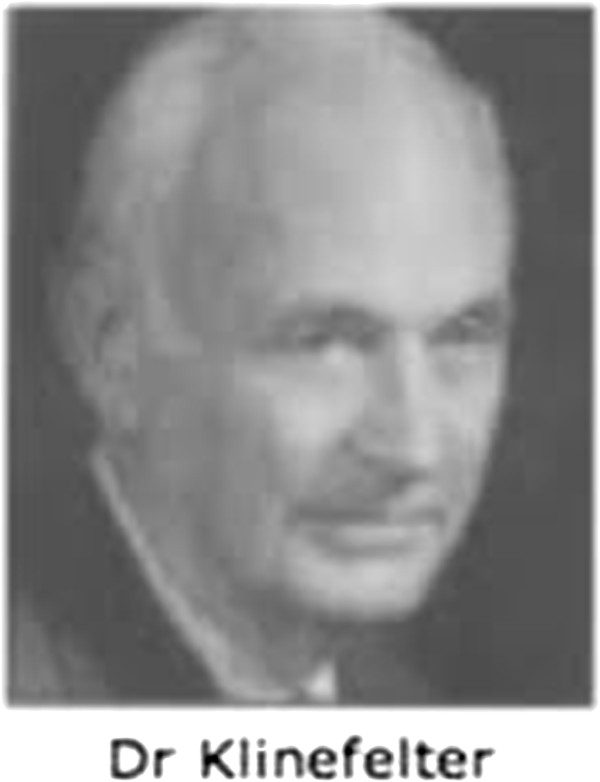
**Harry Fitch Klinefelter, Jr.(1912–1990).**

**Figure 3:**
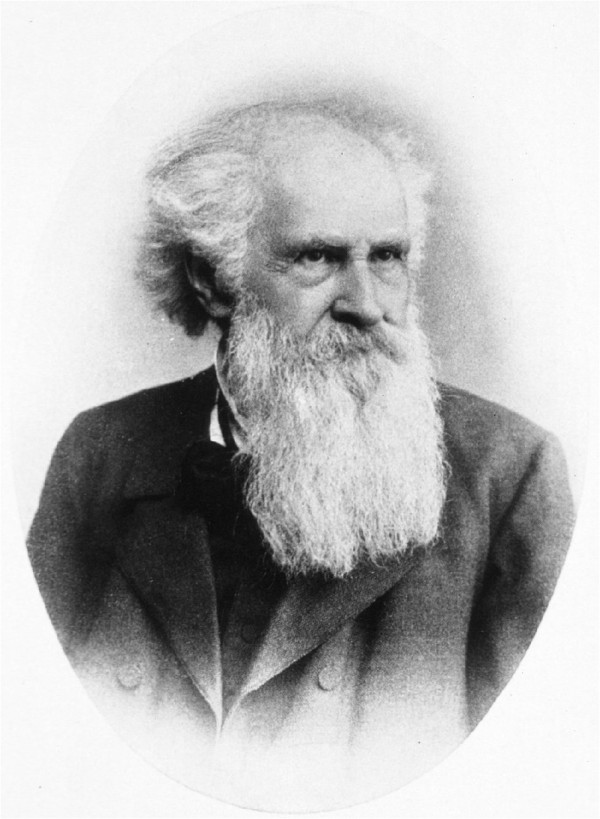
**Franz Leydig (1821 – 1908).**

**Figure 4:**
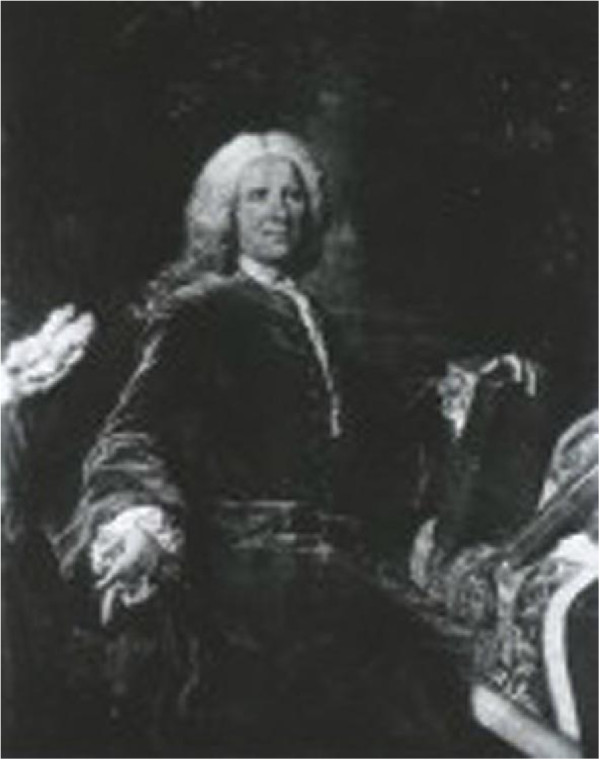
**François Gigot de La Peyronie (1678–1747).**

**Figure 5:**
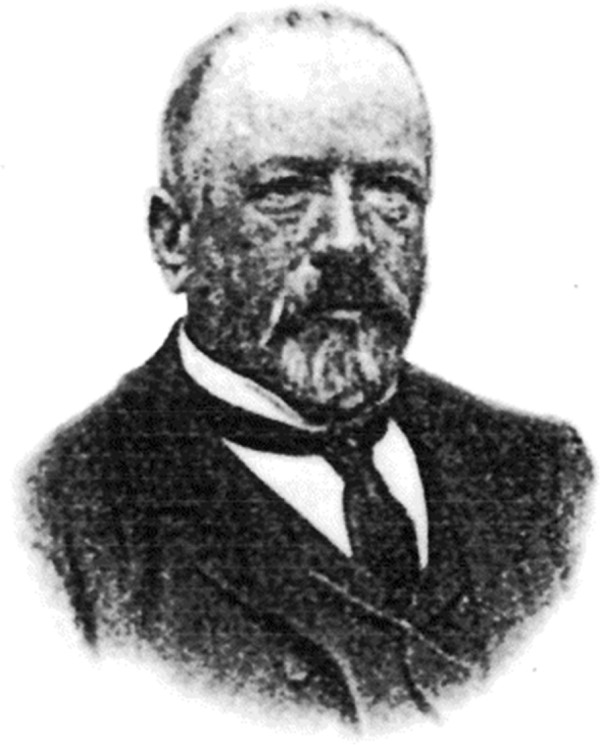
**Enrico Sertoli (1842–1910).**
